# Genome-wide Identification of Metal Tolerance Protein Genes in Peanut: Differential Expression in the Root of Two Contrasting Cultivars Under Metal Stresses

**DOI:** 10.3389/fpls.2022.791200

**Published:** 2022-04-01

**Authors:** Xueqin Wang, Chaohui Wang, Zheng Zhang, Gangrong Shi

**Affiliations:** College of Life Sciences, Huaibei Normal University, Huaibei, China

**Keywords:** peanut, metal tolerance protein, metal translocation, genome-wide identification, gene expression, cultivar difference

## Abstract

Metal tolerance proteins (MTP) are Me^2+^/H^+^(K^+^) antiporters that play important roles in the transport of divalent cations in plants. However, their functions in peanut are unknown. In the present study, a total of 24 *AhMTP* genes were identified in peanut, which were divided into seven groups belonging to three substrate-specific clusters (Zn-CDFs, Zn/Fe-CDFs, and Mn-CDFs). All *AhMTP* genes underwent whole genome or segmental gene duplication events except *AhMTP12*. Most *AhMTP* members within the same subfamily or group generally have similar gene and protein structural characteristics. However, some genes, such as *AhMTP1.3*, *AhMTP2.4*, and *AhMTP12*, showed wide divergences. Most of *AhMTP* genes preferentially expressed in reproductive tissues, suggesting that these genes might play roles in metal transport during the pod and seed development stages. Excess metal exposure induced expressions for most of *AhMTP* genes in peanut roots depending on cultivars. By contrast, *AhMTP* genes in the root of Fenghua 1 were more sensitive to excess Fe, Cd, and Zn exposure than that of Silihong. Stepwise linear regression analysis showed that the percentage of Fe in shoots significantly and positively correlated with the expression of *AhMTP4.1*, *AhMTP9.1*, and *AhMTPC4.1*, but negatively correlated with that of *AhMTPC2.1* and *AhMTP12*. The expression of *AhMTP1.1* showed a significant and negative correlation with the percentage of Mn in shoots. The percentage of Zn in shoots was significantly and positively correlated with the expression of *AhMTP2.1* but was negatively correlated with that of *AhMTPC2.1*. The differential responses of *AhMTP* genes to metal exposure might be, at least partially, responsible for the different metal translocation from roots to shoots between Fenghua 1 and Silihong.

## Introduction

Some divalent metal ions, such as Zn^2+^, Fe^2+^, Mn^2+^, Cu^2+^, Co^2+^, and Ni^2+^, are essential elements in plants, playing crucial roles in numerous processes, including DNA replication, protein processing, photosynthesis, electron transport in the chloroplasts, and mitochondria. Although deficiency of these metals negatively affects plant growth and development, they can also result in toxicity at excessive levels (Bhardwaj et al., 2020). Correspondingly, plants have deployed a variety of adaptive mechanisms to precisely maintain metal homeostasis, including controlling the uptake, efflux, mobilization, translocation, trafficking, and storage (Liu et al., 2019). All these processes are mediated by numerous transporters belonging to different protein families including cation diffusion facilitators (CDFs).

The CDFs have been identified to be Me^2+^/H^+^(K^+^) antiporters that are involved in the transport of divalent cations such Fe^2+^, Zn^2+^, Cd^2+^, Mn^2+^, Ni^2+^, or Co^2+^ in both prokaryotes and eukaryotes (Singh et al., 2015). The CDF family contains three sub-families: (1) Zn-CDF, (2) Fe/Zn-CDFs, and (3) Mn-CDFs (Montanini et al., 2007). Most CDF proteins contain approximately six transmembrane domains (TMDs), a modified signature, and a C-terminal cation efflux domain (Montanini et al., 2007). The six TMDs are normally interconnected by extra-and intra-cellular loops. Among them, one cytosolic loop usually contains a histidine-rich domain, which is predicted to be potential metal binding domains (Montanini et al., 2007). Plant CDFs are usually named metal tolerance proteins (MTPs), while vertebrate CDFs are called solute carrier family 30 (SLC30) or zinc transporter (ZnT).

The MTP proteins in plants can be classified into seven phylogenetic groups: the groups 1 (MTP1–MTP4), 5 (MTP5), and 12 (MTP12) function as Zn-CDFs, the groups 6 (MTP6) and 7 (MTP7) as Fe/Zn-CDFs, and the groups 8 (MTP8) and 9 (MTP9–MTP11) as Mn-CDFs (Gustin et al., 2011). Since the first MTP protein was identified in *Arabidopsis* (ZAT, also named AtMTP1) (van der Zaal et al., 1999), there are large amount of MTP genes were identified in several plant species such as *Arabidopsis* (12 genes), rice (*Oryza sativa*; 10 genes), and wheat (*Triticum aestivum*; 20 genes) (Vatansever et al., 2017). However, most of them have not been functionally characterized in detail. In *Arabidopsis*, both AtMTP1 and AtMTP3 were confirmed to localize in the vacuole, conferring the Zn and/or Co tolerance by the vacuole sequestration of excess Zn^2+^ and/or Co^2+^ (Kobae et al., 2004; Arrivault et al., 2006). AtMTP12 interacts with AtMTP5 to form a functional complex, transporting Zn^2+^ from cytosol to the Golgi apparatus (Fujiwara et al., 2015). As Mn transporters, AtMTP8 and AtMTP11 protect plant cells from Mn toxicity by producing excessive endoplasmic vesicles that phagocytize and excrete Mn^2+^ (Delhaize et al., 2007; Peiter et al., 2007). Rice OsMTP1 localized in vacuole and is involved in the translocation and homeostasis of several divalent metals (Zn^2+^, Cd^2+^, Co^2+^, and Fe^2+^) (Yuan et al., 2012; Menguer et al., 2013). The five Mn-CDF members (OsMTP8.1, OsMTP8.2, OsMTP9, OsMTP11, and OsMTP11.1) were characterized to participate in the maintenance of Mn homeostasis in rice (Chen et al., 2013; Ueno et al., 2015; Takemoto et al., 2017; Ma et al., 2018; Tsunemitsu et al., 2018).

Peanut (*Arachis hypogaea* L., 2*n* = 4*x* = 40) is one of the major oilseed crops grown throughout the tropics and subtropics regions. It provides both edible oil and food protein for people all over the world and accounts for 30% of the total oilseed production in China. Like other crops, peanut often encounters various metal stresses during its life history, which not only limits yield, but also threatens human health due to the accumulation of toxic metals (Liu et al., 2017; Yu et al., 2019). We have demonstrated that peanuts show wide cultivar variation in Cd accumulation and tolerance (Su et al., 2013, 2014); however, the underlying mechanism is not fully understood. Recently, the whole-genome sequences of the cultivated peanut (cv. *Tifrunner*) as well as the two wild ancestral species, *A. duranensis* and *A. ipaënsis*, have been released (Bertioli et al., 2016, 2019). Based on these important resources, genome-wide analysis has been conducted on some gene families such as *WRKY* (Song et al., 2016), *Mlo* (Mildew Locus O; Traore et al., 2021), growth-regulating factors (*GRF*s) (Zhao et al., 2019), and monosaccharide transporter (*MST*) (Wan et al., 2020). However, little progress has been made in peanut *MTP* genes.

To fill the knowledge gap, we identified 24 *AhMTP*s from cultivated peanut and characterized the structure and evolutionary relationship of these genes. Furthermore, two peanut cultivars (Fenghua 1 and Silihong) differing in Cd and Fe-deficiency tolerance (Liu et al., 2017; Cao et al., 2019; Yu et al., 2019) were used for evaluating the expression of *AhMTP* genes in response to several divalent metals (Fe^2+^, Mn^2+^, Zn^2+^, and Cd^2+^). Our findings are expected to provide a perspective on the evolution of *MTP* genes in peanut and were helpful for further functional characterization of *AhMTP* genes, shedding some light on the molecular mechanisms of metal transport and homeostasis in peanut.

## Materials and Methods

### Identification of *MTP* Genes in Peanut

To identify peanut *MTP* genes, the protein sequences of *Arabidopsis* (12 genes) and rice (10 genes) were obtained from phytozome^1^ and were used as queries for TBLASTP against the peanut genome on PeanutBase.^2^ All retrieved MTP protein sequences were examined with the hmmscan tool,^3^ and the candidates containing cation efflux domain (PF01545) were recognized as MTP proteins.

### Phylogenetic Analysis

The MTP protein sequences of peanut, *Arabidopsis*, rice, and cucumber (*Cucumis sativus*) were aligned by clustalw in MEGA-X program (v10.2.6). The aligned files were used to construct a phylogenetic tree of the family members using the with neighbor-joining (NJ) method based on the p-distance model with 1,000 bootstrap replicates. The constructed evolutionary tree was displayed and manipulated using an online software iTOL.^4^

### Physicochemical Properties and Structure Characteristics of AhMTP Proteins

Physiochemical properties of AhMTP proteins including molecular weight (MW), amino acid number, grand average of hydropathicity (GRAVY), instability, aliphatic index, and isoelectric points (pI) were analyzed using ProtParam tool^5^ (Duvaud et al., 2021). TMDs of AhMTP proteins were predicted using TOPCONS^6^ (Tsirigos et al., 2015). Subcellular localization of proteins was predicted using Plant-mPLoc^7^ (Chou and Shen, 2010). The conserved motifs and domains in AhMTP sequences were examined using the MEME v. 5.3.3^8^ and Pfam tool,^9^ respectively (Bailey et al., 2006; Mistry et al., 2020). Homology-modeled 3D structures of AhMTP proteins were predicted using the SwissModel^10^ (Waterhouse et al., 2018).

### Structure, Duplication, and Ka/Ks of *AhMTP* Genes

The exon-intron structure of all *AhMTP* genes was determined using GSDS v. 2.0^11^ (Hu et al., 2015). Gene collinearity and Ka/Ks (ratios of the number of nonsynonymous substitutions per nonsynonymous site to the number of synonymous substitutions per synonymous site) were analyzed by One Step MCScanX and simple Ka/Ks calculator (NJ) of TBtools software, respectively (Chen et al., 2020). Diagrams of exon-intron organization and gene duplication event were drawn using TBtools software (Chen et al., 2020).

### Cis-acting Regulatory Elements and MicroRNA Target Sites of *AhMTP* Genes

The coding and promoter (upstream 1.0 kb) sequences of *AhMTP* genes were retrieved from PeanutBase.^12^ The promoter sequences were used for prediction of cis-acting regulatory elements (CREs) using PlantCARE (Lescot et al., 2002). The coding sequences were used for analyzing miRNA target sites by psRNATarget (Dai et al., 2018).

### Expression Profiles of *AhMTP* Genes in Different Peanut Tissues

Expression profiles of *AhMTP* genes from cv. *Tifrunner* were identified using RNA-seq data obtained from PeanutBase (See footnote 12) (Clevenger et al., 2016). Read counts were transformed to FPKM (fragments per kilobase of exon per million aligned fragments), and the heatmap diagram was constructed with lg^(FPKM + 1)^ using TBtools (Chen et al., 2020).

### Plant Growth, Metal Determination, and qRT-PCR Analysis

Based on our previous studies (Liu et al., 2017; Cao et al., 2019; Yu et al., 2019), two peanut cultivars differing in Cd and Fe-deficiency tolerance, Fenghua 1 (Cd tolerant but sensitive to Fe deficiency), and Silihong (Cd sensitive but tolerant to iron deficiency) were used for determining relationships between the expression of *AhMTP* genes and metal tolerance and accumulation in peanut plants. The two cultivars are widely cultivated in the main production area of peanut in China, having close genetic relationship but differing in metal accumulation, translocation, and tolerance and therefore, being good model plants to study the mechanisms of metal translocation (Liu et al., 2017; Cao et al., 2019; Yu et al., 2019). After surface sterilized with 5% sodium hypochlorite (1 min), seeds were presoaked in distilled water for 24 h and then, they were sown in sand for germination. Three-day-old uniform seedlings were transferred to polyethylene pots and cultured as previously reported (Su et al., 2014). The 10-day-old seedlings were treated with 0.1 mM CdCl_2_, 0.5 mM FeSO_4_, 1 mM MnSO_4_, or 0.5 mM ZnSO_4_ in hydroponic cultures, with those without additional metals as the control. The experiment was arranged in a randomized complete design with triplications (pots) for each treatment. Each replication includes three seedlings. During the growing period, pots were randomly arranged and moved daily for minimizing position effects. After 4 days of metal exposure, plants were harvested and fresh root tissues were sampled for qRT-PCR analysis.

The harvested plants were separated into roots and shoots, and then, the roots were rinsed with 20 mM Na_2_EDTA for 15 min to remove surface-bound metals. After oven-drying, dry weight (DW) of roots and shoots was weighed, and thereafter, tissues were ground into powder. Root (0.1 g) and shoot (0.5 g) samples were digested with HNO_3_–HClO_4_ (3:1, *v*/*v*) as the method described by Su et al. (2014). Concentrations of Fe, Zn, Cd, and Mn were determined by flame atomic absorbance spectrometry (WFX-210, Beijing Rayleigh Analytical Instrument Company, China). Metal translocation from roots to shoots was indicated as the percentage of metal in the shoot, which was calculated as following equation:


$$\begin{array}{l} \mathrm{Percentage of metal in shoots}\;\left(\%\right)=100\times \\ \frac{\mathrm{shoot}\;\mathrm{DW}\times \mathrm{metal conc}.\mathrm{in shoots}}{\left(\begin{array}{l} \mathrm{shoot}\;\mathrm{DW}\times \mathrm{metal conc}.\mathrm{in shoots}+\mathrm{root}\;\mathrm{DW}\times \\ \;\;\;\;\;\;\;\;\;\;\;\;\;\;\;\;\;\;\;\;\;\;\;\;\;\;\;\;\;\;\;\;\;\mathrm{metal conc}.\mathrm{in roots} \end{array}\right)} \end{array}$$

To investigate the expression of *AhMTP* genes in responses to excessive metal stress, the first homolog of each *AhMTP* gene as well as *AhMTP12* was selected for qRT-PCR analysis as the method described previously (Cao et al., 2019), with *Ah60S* as the endogenous reference gene. Primers are listed in Supplementary Table 1. Three technical replications were carried out for each sample. The relative gene expression was calculated using the 2^−ΔΔCT^ method.

### Statistical Analysis

Data were subjected to one-way analysis of variance, and significant variations among means were determined by the Duncan’s Multiple Range Test at a probability level of 5%. Stepwise linear regression analysis was performed on the percentage of metal in shoots and expression of *AhMTP* genes. All statistical analysis was conducted using IBM SPSS Statistics v. 22 (IBM, New York, United States).

## Results

### Summary of the *AhMTP* Gene Family in Peanut

A total of 24 genes were identified in peanut, including three homologous genes of *AhMTP1* (*AhMTP1.1*/*1.2*/*1.3*), four homologous genes of *AhMTP2* (*AhMTP2.1*/*2.2*/*2.3*/*2.4*), two homologous genes of *AhMTP4* (*AhMTP4.1*/*4.2*), two homologous genes of *AhMTP9* (*AhMTP9.1*/*9.2*), two homologous genes of *AhMTP10* (*AhMTP10.1*/*10.2*), four homologous genes of *AhMTP11* (*AhMTP11.1*/*11.2*/*11.3*/*11.4*), two homologous genes of *AhMTPC2* (*AhMTPC2.1*/*C2.2*), two homologous genes of *AhMTPC4* (*AhMTPC4.1*/*C4.2*), two homologous genes of *AhMTPB* (*AhMTPB1*/*B2*), and *AhMTP12* (Table 1). The length of *AhMTP* genes varied from 970 bp (*AhMTP1.3*) to 9,270 bp (*AhMTP2.3*), with CDS lengths from 345 bp (*AhMTP1.3*) to 2,604 bp (*AhMTP12*). The amino acid number of AhMTP proteins ranged from 114 (AhMTP1.3) to 867 (AhMTP12), and the molecular weight of AhMTP proteins varied from 12.20 kDa (AhMTP1.3) to 97.04 kDa (AhMTP12). The instability, aliphatic index, and GRAVY of the AhMTPs ranged from 25.32 (AhMTP1.2) to 48.77 (AhMTP1.3), from 91.56 (AhMTP10.1) to 120.25 (AhMTP2.3), and from-0.221 (AhMTP9.1) to 0.474 (AhMTP2.3), respectively. The isoelectric point (pI) ranged from 4.92 (AhMTP11.3) to 8.67 (AhMTPC4.1), with 15 AhMTP members pI <7 and 9 AhMTP members pI >7 (Table 1). The number of TMDs showed a wide variation among AhMTP proteins, and most AhMTPs contained 4–6 TMDs (Table 1). All AhMTP proteins were predicted to localize to vacuole membranes (Table 1).

**Table 1:** Physicochemical properties and subcellular localization of the 24 metal tolerance proteins (MTPs) identified in peanut.

Gene name	Gene ID	Gene length (bp)	CDS length (bp)	MW (kDa)	aa	Instability	Aliphatic index	GRAVY	PI	TMD	Subcellular localization
*AhMTP1.1*	arahy.ARQ7QI	3,937	1,287	47.14	428	27.63	99.09	−0.064	5.91	6	Vacuole
*AhMTP1.2*	arahy.854BZC	3,902	1,293	47.39	430	25.32	98.63	−0.072	5.95	6	Vacuole
*AhMTP1.3*	arahy.NB6CJE	970	345	12.20	114	48.77	100.18	0.259	5.28	2	Vacuole
*AhMTP2.1*	arahy.5I5ID6	5,188	1,509	54.78	502	42.25	95.62	−0.090	6.75	5	Vacuole
*AhMTP2.2*	arahy.L0WASJ	5,250	1,509	54.80	502	43.11	95.24	−0.098	6.67	5	Vacuole
*AhMTP2.3*	arahy.INQ3MB	9,270	828	30.33	275	26.19	120.25	0.474	8.15	6	Cell/Vacuole membrane
*AhMTP2.4*	arahy.XK0FMC	3,180	1,059	39.83	352	44.40	99.72	0.170	6.72	3	Vacuole
*AhMTP4.1*	arahy.N1ZWYI	4,890	1,266	47.34	421	46.25	108.86	0.005	5.87	5	Vacuole
*AhMTP4.2*	arahy.LH0XEN	5,012	1,266	47.38	421	46.32	108.86	0.000	5.93	5	Vacuole
*AhMTP9.1*	arahy.1E45SP	8,680	1,245	47.62	414	45.50	93.53	−0.221	8.20	4	Vacuole
*AhMTP9.2*	arahy.3T6XNU	5,968	801	30.45	266	38.28	104.14	0.056	6.31	4	Vacuole
*AhMTP10.1*	arahy.DW8A2N	4,985	1,236	46.78	411	43.20	91.56	−0.109	7.24	6	Cell/Vacuole membrane
*AhMTP10.2*	arahy.JR9DQ2	4,952	1,236	47.01	411	46.26	91.56	−0.129	8.64	6	Cell/Vacuole membrane
*AhMTP11.1*	arahy.J9LUB0	5,202	1,191	44.99	396	47.43	100.71	−0.009	4.97	6	Vacuole
*AhMTP11.2*	arahy.C1W7MD	4,156	1,215	46.04	404	45.81	101.36	0.008	5.04	6	Vacuole
*AhMTP11.3*	arahy.F3QZDX	4,293	1,191	44.97	396	46.11	100.71	−0.011	4.92	6	Cell/Vacuole membrane
*AhMTP11.4*	arahy.HJ8JHV	5,202	1,191	44.99	396	47.43	100.71	−0.009	4.97	6	Vacuole
*AhMTP12*	arahy.LDE0EK	2,605	2,604	97.04	867	43.83	97.01	0.033	7.20	16	Vacuole
*AhMTPB1*	arahy.IEEP0H	1,165	1,164	42.85	387	35.35	109.53	0.199	5.89	6	Vacuole
*AhMTPB2*	arahy.WGY3ZU	1,168	1,158	42.47	385	35.86	111.38	0.246	5.83	6	Vacuole
*AhMTPC2.1*	arahy.C1QR5S	5,448	1,143	42.39	380	43.04	98.50	0.149	8.33	5	Vacuole
*AhMTPC2.2*	arahy.F8DN54	5,482	1,143	42.39	380	43.04	98.50	0.149	8.33	5	Vacuole
*AhMTPC4.1*	arahy.2A2X48	4,032	1,329	49.01	442	33.62	94.66	0.005	8.67	4	Vacuole
*AhMTPC4.2*	arahy.660VQ8	4,544	1,326	48.88	441	33.48	94.88	−0.005	7.73	6	Vacuole

*MW, aa, GRAVY, PI, and TMD refer to molecular weight, amino acid number, grand average of hydropathicity, isoelectric points, and transmembrane domain, respectively*.

### Phylogenetic Analysis of MTP Gene Families

**Figure 1:**
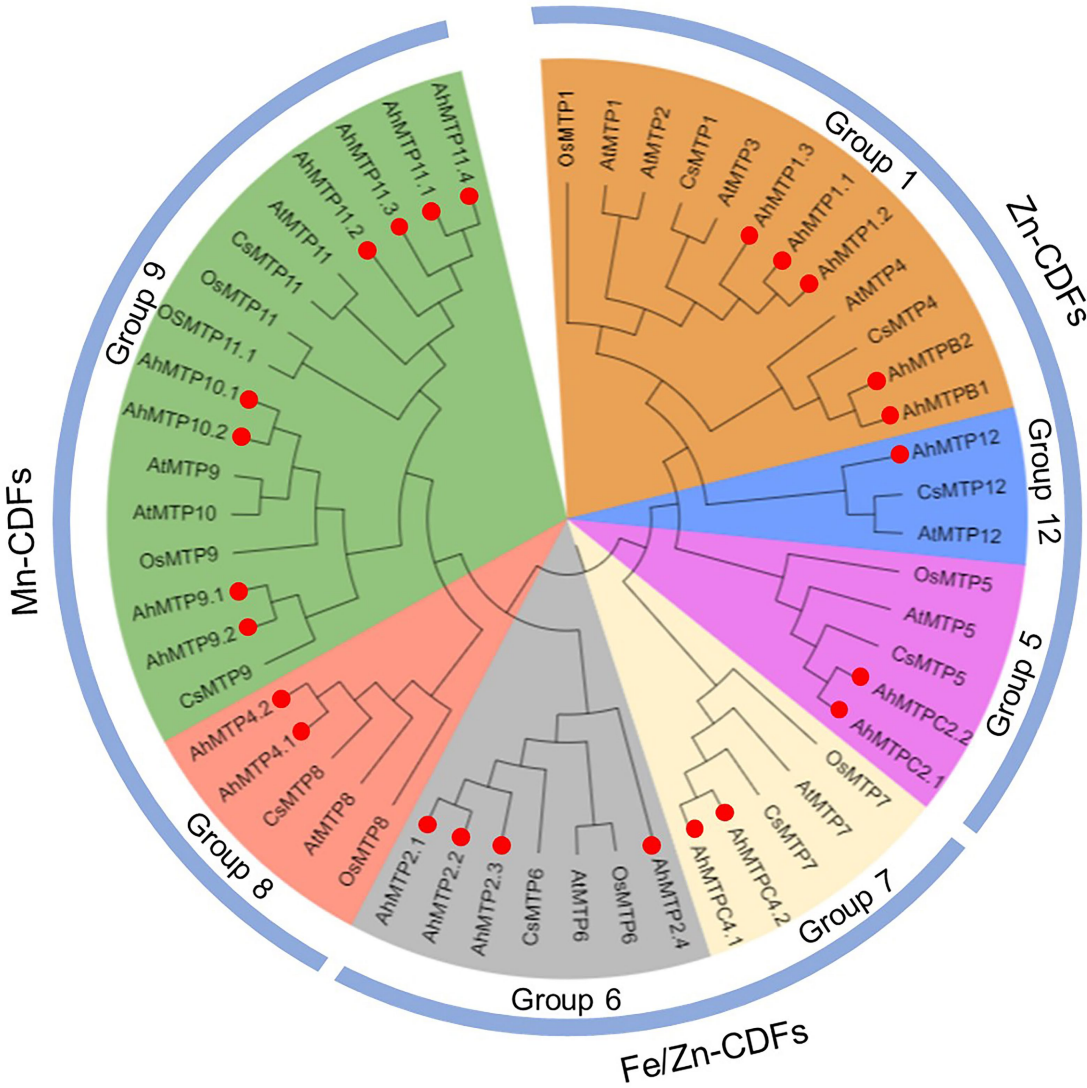
Phylogenetic relationships of MTP proteins in peanut and other plant species. Red solid circles represent the 24 AhMTP proteins of peanut.

Phylogenetic relationship of 53 MTPs from peanut, *Arabidopsis*, rice, and cucumber was analyzed with the NJ method. These MTP proteins were divided into seven groups (1, 5, 6, 7, 8, 9, and 12), belonging to three major sub-families (Zn-CDFs, Zn/Fe-CDFs, and Mn-CDFs; Figure 1). Of the seven primary groups, group 9 is the largest one containing eight AhMTPs (AhMTP9.1/9.2, AhMTP10.1/10.2, and AhMTP11.1/11.2/11.3/11.4), followed by group 1 (AhMTP1.1/1.2/1.3 and AhMTPB1/B2) and 6 (AhMTP2.1/2.2/2.3/2.4), while group 12 is the smallest group with only one member (AhMTP12). The remaining three groups contained two AhMTP members each (Figure 1).

### Structure and Duplication of *AhMTP* Genes

**Figure 2:**
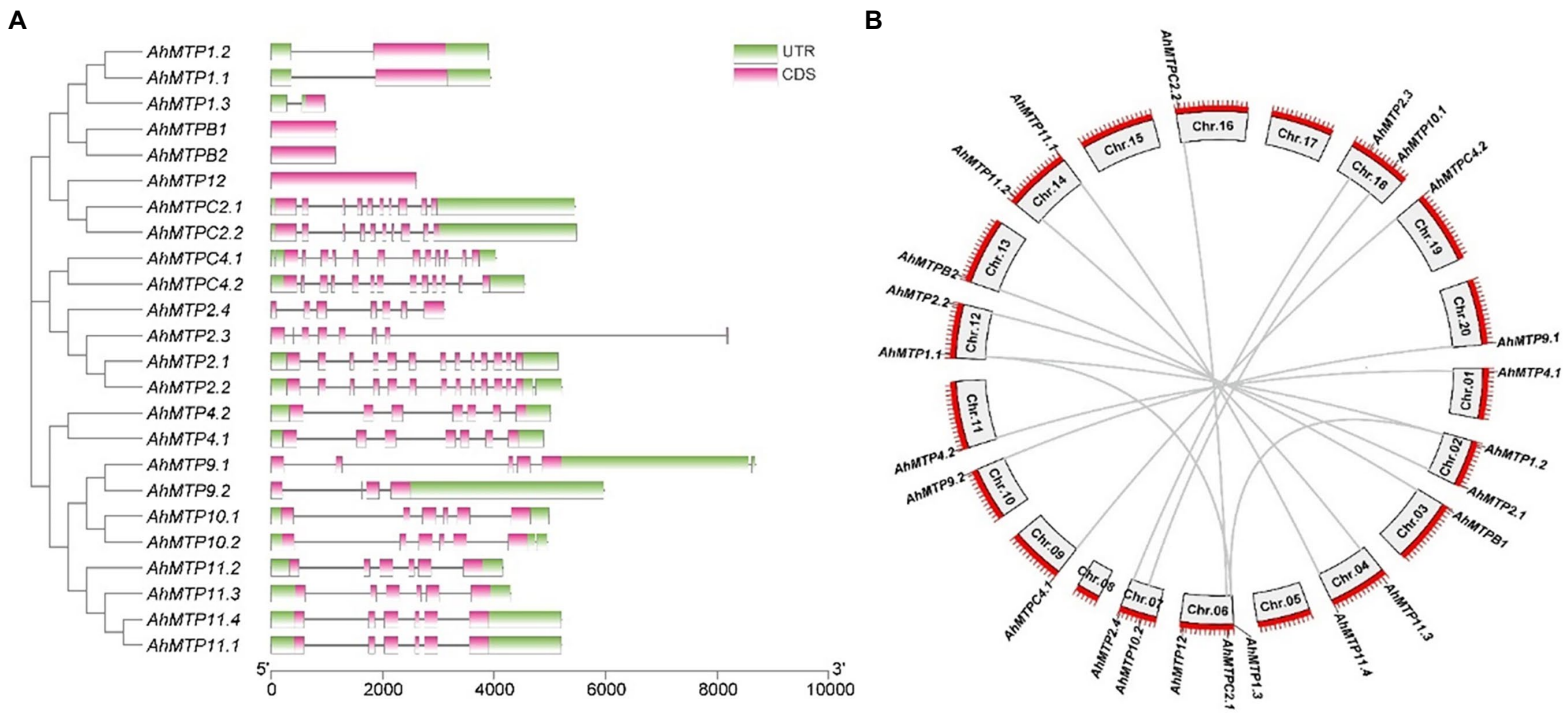
Phylogenetic relationships, exon-intron organization, and collinearity analysis of AhMTP genes from peanut. **(A)** Phylogenetic tree and exon-intron structure of *AhMTP* genes. **(B)** Chromosomal locations and duplications of *AhMTP* genes.

To gain insight into the evolution of the *MTP* family in peanut, exon-intron organizations of *AhMTP* genes were examined. As showed in Figure 2A, *AhMTP* genes belonging to the same groups showed similar exon-intron organizations, which coincided with the results obtained from the phylogenetic analysis. The group 1 and 12 of Zn-CDFs contained only one exon (with one or without intron), whereas group 5 possessed 10 exons (nine intron). Zn/Fe-CDF genes contained 12–13 exons except *AhMTP2.3*/*2.4*, which harbored seven exons. The group 8 of Mn-CDFs possessed seven exons, while most genes of group 9 contained six exons except *AhMTP9.1* and *AhMTP9.2*, in which five and four exons were harbored, respectively.

*AhMTP* genes were located unevenly in 16 chromosomes. A total of 13 and 11 *AhMTP* genes were identified from the subgenome A (Chr.01–10) and B (Chr.11–20), respectively (Figure 2B). The Chr.06 contained three *AhMTP* genes, the Chr. 02, 04, 07, 12, 14, and 18 had two genes each, each of the nine chromosomes (01, 03, 09, 10, 11, 13, 16, 19, and 20) contained only one gene, while no *AhMTP* gene was identified in the Chr. 05, 08, 15, and 17. All *AhMTP* genes that located in the same chromosome showed greatly large distances (Figure 2B).

Collinearity analysis revealed that almost all *AhMTP* genes experienced gene duplication events except *AhMTP12*, resulting in 13 gene pairs (Figure 2B). Interestingly, all the 11 *AhMTP* genes of the subgenome B were crossly collineared with corresponding genes of subgenome A, forming 11 gene pairs including *AhMTPC4.1/C4.2*, *AhMTP9.1/9.2*, *AhMTP2.1/2.2*, *AhMTP1.1/1.2*, *AhMTPC2.1/C2.2*, *AhMTP11.3/11.2*, *AhMTP11.4/11.1*, *AhMTPB1/B2*, *AhMTP10.1/10.2*, *AhMTP4.1/4.2*, and *AhMTP2.4/2.3*. These collinear blocks result from whole-genome duplications (WGDs). In addition, *AhMTP1.3* was also identified to collinear with *AhMTP1.1* and *AhMTP1.2*, respectively. The pair of *AhMTP1.2/1.3* might be result from segmental duplication because they are in different chromosomes of the same subgenome. No tandem duplication was detected in *AhMTP* genes. The Ka/Ks ratios of all gene duplication pairs were less than 1 (Table 2), indicating that *AhMTP* genes evolved under purifying selection (Hurst, 2002).

**Table 2:** Ka/Ks analysis of all gene duplication pairs for *AhMTP* genes.

Duplicated pair	Duplicate type	Ka	Ks	Ka/Ks	Positive selection
*AhMTPC4.1*/*C4.2*	Whole-genome	0.005	0.023	0.231	No
*AhMTP9.2*/*9.1*	Whole-genome	0.137	0.210	0.650	No
*AhMTP2.1*/*2.2*	Whole-genome	0.003	0.023	0.113	No
*AhMTP1.2*/*1.3*	Segmental	0.107	0.711	0.150	No
*AhMTP1.2*/*1.1*	Segmental	0.008	0.058	0.141	No
*AhMTPC2.1*/*C2.2*	Whole-genome	0.000	0.041	0.000	No
*AhMTP11.3*/*11.2*	Whole-genome	0.007	0.013	0.563	No
*AhMTP11.4*/*11.1*	Whole-genome	0.000	0.000	NaN	No
*AhMTPB1*/*B2*	Whole-genome	0.008	0.028	0.286	No
*AhMTP10.2*/*10.1*	Whole-genome	0.005	0.026	0.206	No
*AhMTP4.1*/*4.2*	Whole-genome	0.003	0.037	0.086	No
*AhMTP1.3*/*1.1*	Whole-genome	0.116	0.682	0.170	No
*AhMTP2.4*/*2.3*	Whole-genome	0.480	0.558	0.859	No

### Conserved Motifs, Domain Architectures, and Models of AhMTP Proteins

**Figure 3:**
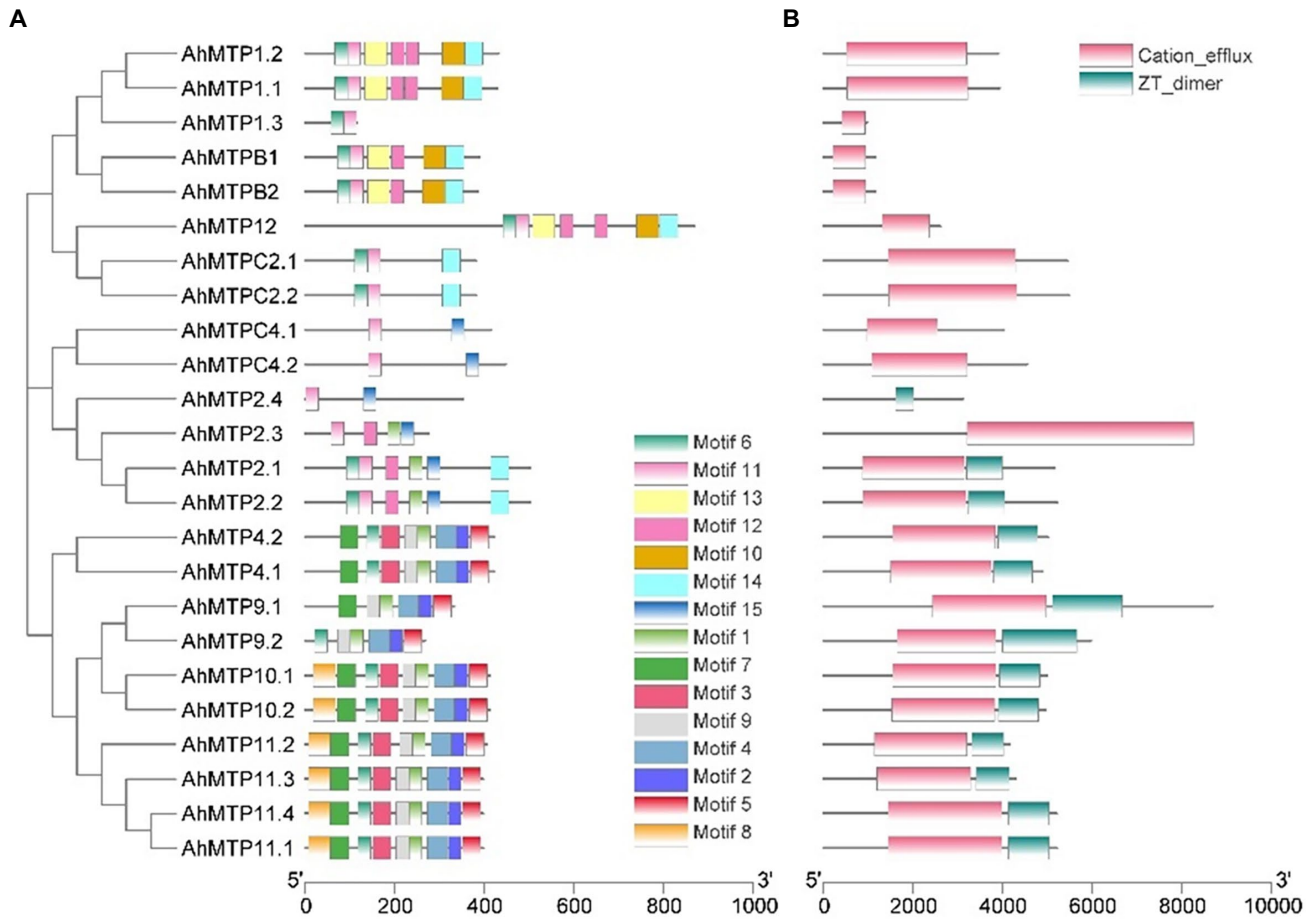
Distributions of the conserved motifs **(A)** and domains **(B)** in AhMTP proteins from peanut.

AhMTP proteins contained a total of 15 conserved motifs, among them, eight motifs (3, 4, 5, 6, 10, 11, 13, and 14) were annotated to be the TMD or CTD of cation_efflux domains according to the InterProScan tools (Figure 3A and Supplementary Table 2). It was observed that conserved motifs were specifically distributed in the members of different cluster or group (Figure 3A). Two motifs (6 and 11) were shared by eight AhMTP proteins belonging to the Zn-CDF cluster. Interestingly, 10 members of the Mn-CDF cluster contained a specific motif complex composed of motifs 1, 2, 4, 5, and 9. The six proteins of the Fe/Zn-CDF subfamily widely varied in motif distribution and shared two motifs (motif 11 and 15; Figure 3A).

Conserved domain analysis revealed that all AhMTP proteins except AhMTP2.4 contained the typical domain of MTP, cation efflux domain (Figure 3B). Additionally, a dimerization domain of zinc transporters, ZT-dimer, was also identified in all members of Mn-CDF cluster and three members (AhMTP2.1/2.2/2.4) of group 6 belonging to Fe/Zn-CDFs (Figure 3B).

To better understand the functions of AhMTP proteins, their sequences were modeled using the SwissModel. As showed in Table 3, the sequence identity was ranged from 16.25 to 42.86%, the value of GMQE ranged from 0.19 to 0.53, and QMEANDisCo global score ranged from 0.41 to 0.70. These data suggested a high quality of a 3D protein structure models of AhMTP proteins, which were presented as Supplementary Figure 1. The 3D-model prediction revealed that the three subfamily of AhMTPs differed from each other in the protein structure (Table 3). All members of Zn-CDFs are best modeled with the template, 6xpd.1 (Cryo-EM structure of human ZnT8 double mutant—D110N and D224N, determined in outward-facing conformation). The best model for all Mn-CDF members was 3h90.1 (Structural basis for the autoregulation of the zinc transporter YiiP). Zn/Fe-CDFs were commendably modeled to 5vrf.1 (Cryo-EM structure of the Zinc transporter YiiP from helical crystals) except AhMTP2.4, of which the best model was 3h90.1.

**Table 3:** The best templates of peanut AhMTP proteins selected from the SwissModel template library for building 3D structure models.

Protein name	Template	Sequence identity (%)	Coverage	GMQE	QMEANDisCo Global	Description
MTP1.2	6xpd.1	36.86	A44-430	0.49	0.61 ± 0.05	Zinc transporter 8
MTP1.1	6xpd.1	36.86	A44-428	0.49	0.61 ± 0.05	Zinc transporter 8
MTP1.3	6xpd.1	42.86	A37-113	0.47	0.70 ± 0.11	Zinc transporter 8
MTPB1	6xpd.1	32.18	A53-387	0.53	0.62 ± 0.05	Zinc transporter 8
MTPB2	6xpd.1	32.53	A53-385	0.52	0.61 ± 0.05	Zinc transporter 8
MTPC2.1	6xpd.1	16.25	A94-379	0.44	0.54 ± 0.05	Zinc transporter 8
MTPC2.2	6xpd.1	16.25	A94-379	0.44	0.54 ± 0.05	Zinc transporter 8
MTP12	6xpd.1	24.83	A421-866	0.19	0.49 ± 0.05	Zinc transporter 8
MTPC4.1	5vrf.1	17.36	A96-408	0.33	0.41 ± 0.05	Cadmium and zinc efflux pump FieF
MTPC4.2	5vrf.1	17.48	A95-440	0.36	0.43 ± 0.05	Cadmium and zinc efflux pump FieF
MTP2.1	5vrf.1	27.62	A75-385	0.37	0.55 ± 0.05	Cadmium and zinc efflux pump FieF
MTP2.2	5vrf.1	27.62	A75-385	0.37	0.54 ± 0.05	Cadmium and zinc efflux pump FieF
MTP2.3	5vrf.1	24.86	A41-259	0.39	0.50 ± 0.06	Cadmium and zinc efflux pump FieF
MTP2.4	3h90.1	20.00	A2-246	0.33	0.41 ± 0.05	Ferrous-iron efflux pump fieF
MTP11.4	3h90.1	19.71	A106-386	0.40	0.49 ± 0.05	Ferrous-iron efflux pump fieF
MTP11.1	3h90.1	19.71	A106-386	0.40	0.49 ± 0.05	Ferrous-iron efflux pump fieF
MTP11.3	3h90.1	19.27	A106-387	0.40	0.50 ± 0.05	Ferrous-iron efflux pump fieF
MTP11.2	3h90.1	18.91	A106-395	0.39	0.48 ± 0.05	Ferrous-iron efflux pump fieF
MTP9.1	3h90.1	18.06	A89-322	0.38	0.47 ± 0.05	Ferrous-iron efflux pump fieF
MTP9.2	3h90.1	18.62	A8-256	0.49	0.45 ± 0.05	Ferrous-iron efflux pump fieF
MTP10.1	3h90.1	18.91	A122-402	0.38	0.49 ± 0.05	Ferrous-iron efflux pump fieF
MTP10.2	3h90.1	19.27	A122-402	0.38	0.49 ± 0.05	Ferrous-iron efflux pump fieF
MTP4.2	3h90.1	21.74	A124-405	0.36	0.50 ± 0.05	Ferrous-iron efflux pump fieF
MTP4.1	3h90.1	21.74	A124-405	0.36	0.49 ± 0.05	Ferrous-iron efflux pump fieF

### CREs and the MicroRNA Target Sites of *AhMTP* Genes

To explore the probable post-transcriptional regulation of the *AhMTP* genes, their CREs and microRNA target sites were predicted. A total of 1,548 CREs were identified in the promoter of *AhMTP* genes, and most of them were identified to associate with gene transcription (1,246), light response (155), phytohormonal response (83), and abiotic stress (40) (Table 4 and Supplementary Table 3). The promoter of all *AhMTP* genes harbored gene transcription (CAAT and TATA-box) and light-responsive elements whose number ranged from 12 to 110 and from 1 to 16, respectively. Most of *AhMTP* genes have phytohormone responsive elements in the promoters except *AhMTP1.1*, *AhMTP1.2*, *AhMTP2.4*, *AhMTPC2.1*, and *AhMTPC4.1*. Nineteen *AhMTP* genes were identified to contain biotic stress elements, including ARE (*AhMTP1.1/1.2/1.3*, *AhMTP2.1/2.2*, *AhMTPC2.1/C2.2*, *AhMTP11.1/11.3/11.4*, *AhMTP9.2*, *AhMTP10.1/10.2*, *AhMTP12*), LTR (*AhMTP1.1/1.2*, *AhMTP9.2*, *AhMTP10.1/10.2*, and *AhMTPC4.1/C4.2*), MBS (*AhMTP1.1*/*1.2*, *AhMTPC2.1*, *AhMTP12*, and *AhMTP4.1*), and TC-rich repeats (*AhMTPC2.1*, *AhMTP1.3*, and *AhMTP2.2*).

**Table 4:** Frequency and function of cis-regulatory elements (CREs) in the promoter regions of *AhMTP* genes.

Gene name	Gene transcription	Abiotic stress	Biotic stress	Tissue expression	Secondary metabolism	Phytohormonal response	Light response	Circadian control	Site-binding	Cell-cycle regulation
*AhMTP1.1*	46	4	0	0	0	0	2	0	0	1
*AhMTP1.2*	44	4	0	0	0	0	2	0	0	0
*AhMTP1.3*	37	2	0	0	0	7	8	0	1	0
*AhMTP2.1*	61	1	0	0	1	5	10	0	0	0
*AhMTP2.2*	68	2	0	0	1	7	9	0	0	0
*AhMTP2.3*	29	0	0	0	0	3	15	1	1	1
*AhMTP2.4*	41	0	1	1	0	0	7	0	0	0
*AhMTP4.1*	46	1	1	1	1	2	8	0	0	0
*AhMTP4.2*	110	0	0	0	0	2	3	1	0	0
*AhMTP9.1*	86	0	0	0	1	6	16	0	1	0
*AhMTP9.2*	75	0	0	0	0	1	6	0	0	0
*AhMTP10.1*	49	2	0	1	0	4	7	0	0	0
*AhMTP10.2*	45	3	0	0	1	7	8	0	0	0
*AhMTP11.1*	53	3	0	0	0	9	9	0	1	0
*AhMTP11.2*	25	0	0	1	0	4	4	0	2	0
*AhMTP11.3*	56	1	0	0	0	3	3	0	1	0
*AhMTP11.4*	53	3	0	0	0	9	9	0	1	0
*AhMTP12*	12	2	0	0	0	4	6	0	0	1
*AhMTPB1*	55	0	0	0	0	5	3	0	0	0
*AhMTPB2*	56	0	0	0	0	3	6	0	0	0
*AhMTPC2.1*	42	6	0	0	0	0	1	0	0	0
*AhMTPC2.2*	43	3	0	0	0	1	4	0	0	0
*AhMTPC4.1*	59	1	0	0	0	0	5	0	0	0
*AhMTPC4.2*	55	2	0	0	0	1	4	0	0	0

A total of five miRNAs were identified, with eight genes belonging to three groups as target genes (Table 5). The UPE varied from 14.877 (*ahy-miR156a*/*AhMTPC2.1*) to 21.179 (*ahy-miR3519*/*AhMTP11.2*). Two gene pairs of group 9, *AhMTP9.1/9.2* and *AhMTP11.2/11.3*, were predicted to be targets of *ahy-miR167-5p* and *ahy-miR3519*, respectively. The two members of group 5, *AhMTPC2.1* and *AhMTPC2.2*, were potential target genes of *ahy-miR156a* and *ahy-miR156c*. Possible target sites of *ahy-miR3511-3p* were identified in two genes of group 8, *AhMTP4.1* and *AhMTP4.2*. All identified miRNA targeted genes were inhibited by the corresponding miRNA in a cleavage manner.

**Table 5:** Prediction of miRNAs for *AhMTP* transcripts.

miRNA	Target	Expectation	UPE	miRNA aligned	Target aligned	Inhibition
*ahy-miR167-5p*	*AhMTP9.1*	4.5	16.384	UGAAGCUGCCA GCAUGAUCUU	GGGAUUAUGGU GGCAGCAUCU	Cleavage
*ahy-miR167-5p*	*AhMTP9.2*	4.5	16.874	UGAAGCUGCCA GCAUGAUCUU	GGGAUUAUGGU GGCAGCAUCU	Cleavage
*ahy-miR3519*	*AhMTP11.2*	4.5	21.179	UCAAUCAAUGA CAGCAUUUCA	GCAAAUUAUGU CGAUGAUUGG	Cleavage
*ahy-miR3519*	*AhMTP11.3*	4.5	19.995	UCAAUCAAUGA CAGCAUUUCA	GCAAAUUAUGU CGAUGAUUGG	Cleavage
*ahy-miR156a*	*AhMTPC2.1*	5	14.877	UGACAGAAGAG AGAGAGCAC	AUGCCCUGUUU CUUUUGUUU	Cleavage
*ahy-miR156a*	*AhMTPC2.2*	5	15.246	UGACAGAAGAG AGAGAGCAC	AUGCCCUGUUU CUUUUGUUU	Cleavage
*ahy-miR156c*	*AhMTPC2.1*	5	15.19	UUGACAGAAGA GAGAGAGCAC	AUGCCCUGUUU CUUUUGUUUA	Cleavage
*ahy-miR156c*	*AhMTPC2.2*	5	15.344	UUGACAGAAGA GAGAGAGCAC	AUGCCCUGUUU CUUUUGUUUA	Cleavage
*ahy-miR3511-3p*	*AhMTP4.1*	5	20.147	UGUUACUAUGG CAUCUGGUAA	GAAGUCGAUGC CAUAGUGGCA	Cleavage
*ahy-miR3511-3p*	*AhMTP4.2*	5	16.76	UGUUACUAUGG CAUCUGGUAA	GAAGUUGAUGC CAUAGUGGCA	Cleavage

### Expression Profiles of *AhMTP* Genes in Different Tissues of Peanut

**Figure 4:**
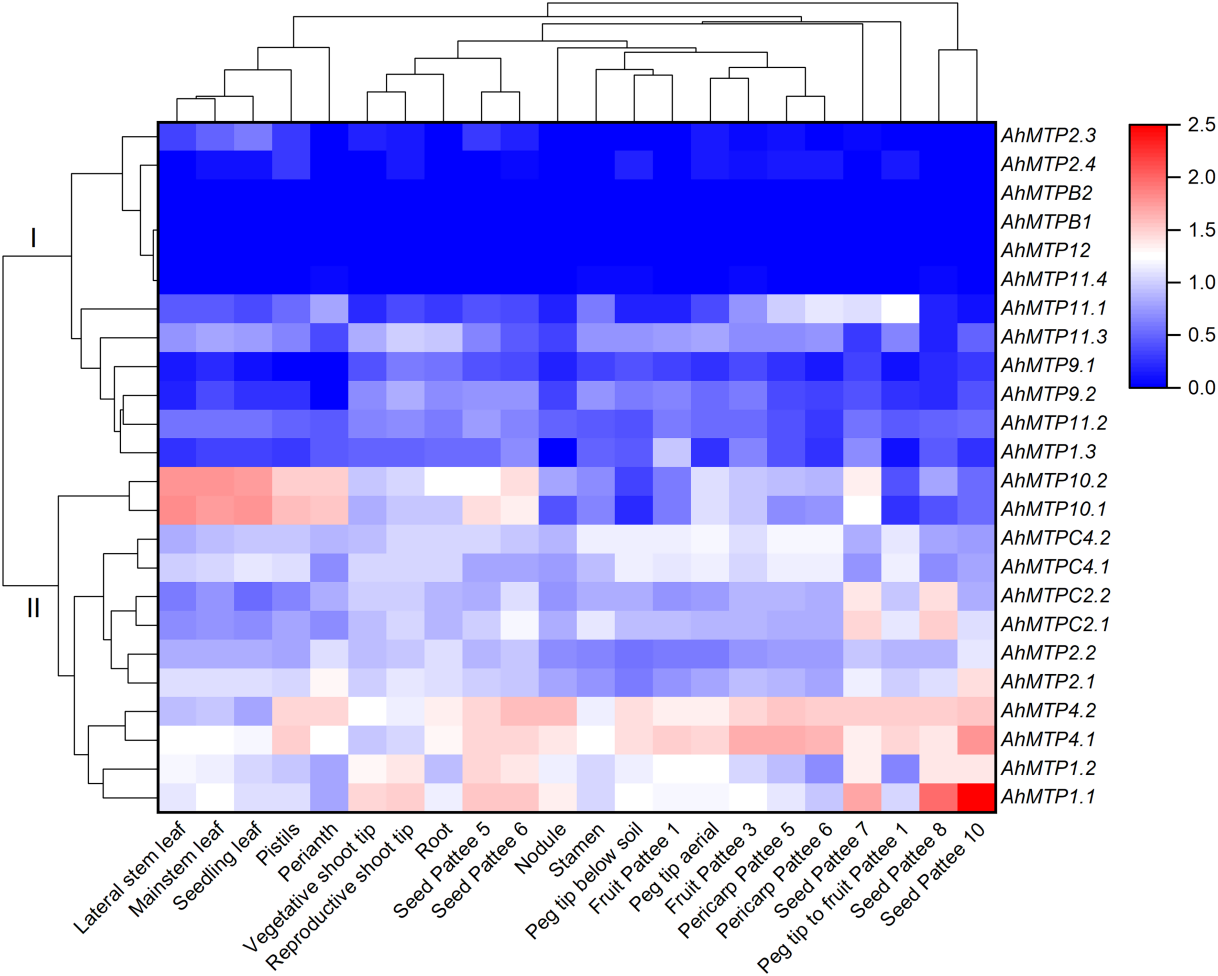
The expression profiles of *AhMTP* genes in different tissues of peanut. Gene expression is expressed in lg^(FPKM + 1)^ and *Z* score normalized. Pattee 1, 3, 5, 6, 7, 8, and 10 represent different developmental stages of peanut pods according to Pattee’ s classification (Clevenger et al., 2016).

RNA-seq data showed that most of the *AhMTP* genes expressed in peanut tissues except *AhMTPB1*, *AhMTPB2*, and *AhMTP12*, which did not express in all the 22 tissues (Supplementary Table 4). To better understand the gene expression profiles, a hierarchical cluster analysis was carried out. As presented in Figure 4, 24 *AhMTP* genes were divided into two clusters: cluster I and II. Cluster I includes 12 genes with low expression levels. Among them, six genes showed very low expression (*AhMTP2.3*/*2.4* and *AhMTP11.4*) or did not express (*AhMTPB1*/*B2* and *AhMTP12*) in any tissues. The other six genes exhibit tissue-specific expressions with low levels. *AhMTP11.1* mainly expressed in peg tip to fruit, seed, and pericarp; *AhMTP11.3* expressed in all tissues but relatively higher in shoot tips and roots, and *AhMTP1.3* mainly expressed in fruit.

Cluster II consists of six pair of *AhMTP* genes including *AhMTP1.1*/*1.2*, *AhMTP2.1*/*2.2*, *AhMTP4.1*/*4.2*, *AhMTPC2.1*/C*2.2*, *AhMTPC4.1*/C*4.2*, and *AhMTP10.1*/*10.2*, with high expression levels in approximately all tissues. Each pair of genes showed a similar expression pattern. *AhMTP1.1*/*1.2* is specifically and highly expressed in shoot tips and seed. *AhMTP4.1*/*4.2* highly expressed in roots, nodules, stamens, peg tips, fruit, peg tip to fruit, pericarp, and seed. *AhMTP10.1*/*10.2* highly expressed in leaves, perianth, and pistil. The highest expression of *AhMTPC2.1*/C*2.2* was in seed.

### Differential Expression of *AhMTP* Genes in the Root of Two Peanut Cultivars

**Figure 5:**
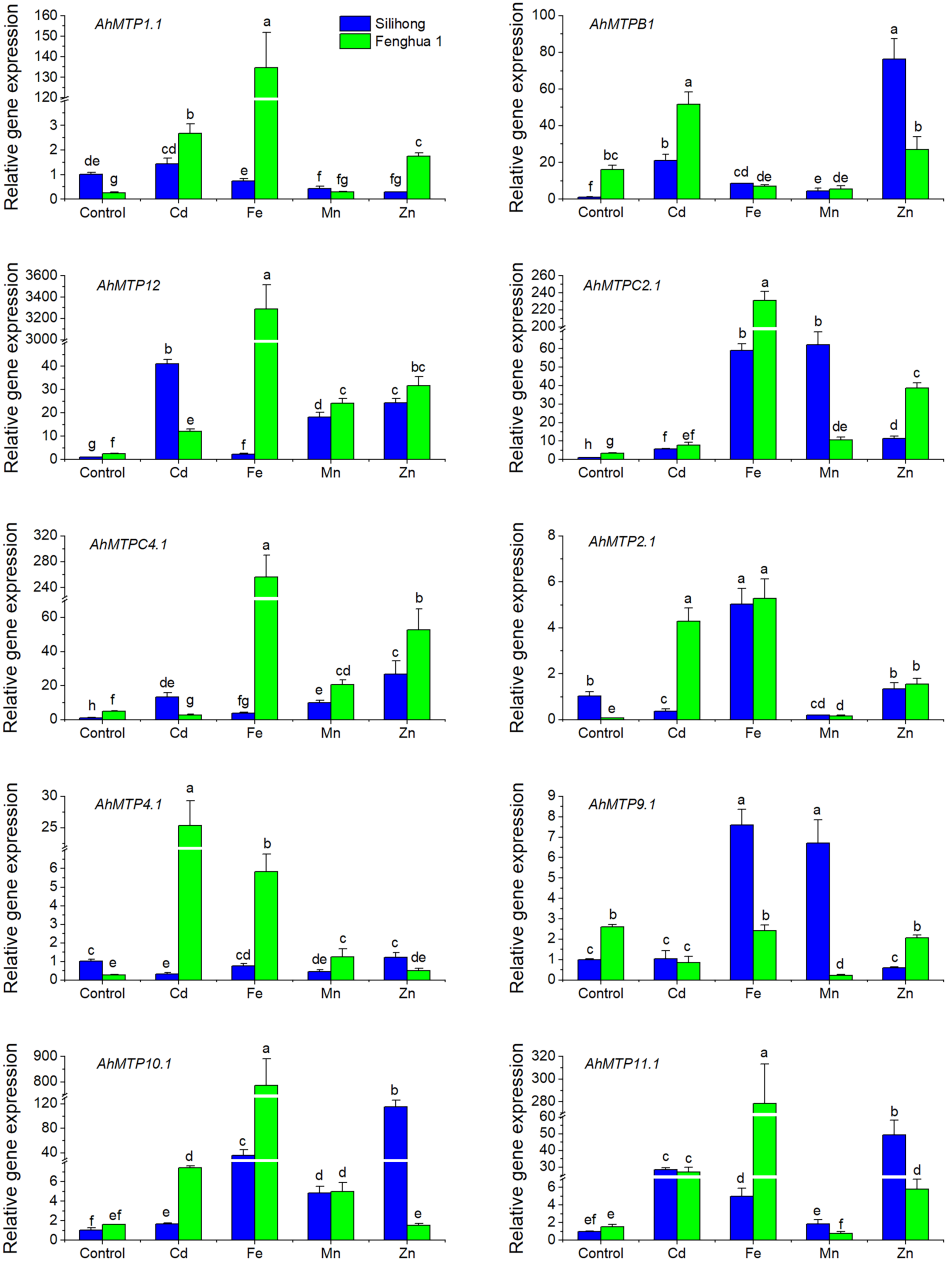
Expression levels of 10 *AhMTP* genes in the root of two peanut cultivars exposed to excess metals. Data (means ± SE, *n* = 3) shared the same letter(s) above the error bars are not significantly different at the 0.05 level by Duncan multiple range test.

To investigate the expression of *AhMTP* genes in responses to excessive metal stress, the first homolog of each *AhMTP* gene as well as *AhMTP12* was selected for qRT-PCR analysis. The two cultivars differ from each other in the expression of *AhMTP* genes as well as their responses to excessive metal stress (Figure 5). Under normal nutrition condition, Fenghua 1 showed higher expressions of *AhMTPB1*, *AhMTP9.1*, *AhMTP12*, *AhMTPC2.1*, and *AhMTPC4.1* than Silihong, while the expression of *AhMTP1.1*, *AhMTP2.1*, and *AhMTP4.1* was higher in Silihong than in Fenghua 1. Cd enhanced the expression of *AhMTPB1*, *AhMTP12*, *AhMTPC2.1*, *AhMTP10.1*, and *AhMTP11.1* for both cultivars, while other genes showed different responses to Cd exposure between the two cultivars. By contrast, Cd induced increase of gene expression was more pronounced in Fenghua 1 than that in Silihong. Although Cd induced expressions for most *AhMTP* genes, some genes such as *AhMTP9.1* and *AhMTPC4.1* in Fenghua 1 as well as *AhMTP2.1* and *AhMTP4.1* in Silihong were observed to be repressed by Cd, while *AhMTP9.1* and *AhMTP1.1* in Silihong remained unaffected.

Excess Fe upregulated the expression of *AhMTP12*, *AhMTPC2.1*, *AhMTPC4.1*, *AhMTP2.1*, *AhMTP10.1*, and *AhMTP11.1* for both cultivars (Figure 5). The induction was more pronounced in Fenghua 1 than that in Silihong for most genes. *AhMTP1.1* and *AhMTP4.1* were significantly induced by Fe stress in Fenghua 1, whereas in Silihong, expressions of these genes were unchanged. *AhMTP9.1* and *AhMTPB1* were induced by Fe stress in Silihong but were unchanged or downregulated in Fenghua 1 (Figure 5).

Excess Mn increased the expression of *AhMTP12*, *AhMTPC4.1*, *AhMTPC2.1*, and *AhMTP10.1* for both cultivars (Figure 5). The expression of *AhMTPB1* and *AhMTP9.1* was increased by Mn in Silihong; however, it was repressed in Fenghua 1. Conversely, the expression of *AhMTP1.1*, *AhMTP2.1*, and *AhMTP4.1* was repressed by excess Mn in Silihong, while in Fenghua 1, they were unaffected. Mn exposure downregulated the expression of *AhMTPB1* in Fenghua but not in Silihong.

Excess Zn induced the expression of *AhMTP11.1*, *AhMTP12*, *AhMTPC2.1*, and *AhMTPC4.1* but repressed that of *AhMTP9.1* for both cultivars, while *AhMTP4.1* was unaffected (Figure 5). The remaining four *AhMTP* genes exhibit cultivar-specific patterns in response to Zn stress. *AhMTP1.1* and *AhMTP2.1* were induced by Zn in Fenghua 1 but were unaffected or repressed in Silihong. Zn dramatically increased the expression of *AhMTPB1* and *AhMTP10.1* in Silihong but did not affect that in Fenghua 1 (Figure 5).

### Differential Metal Translocation in Plants of Two Peanut Cultivars

**Figure 6:**
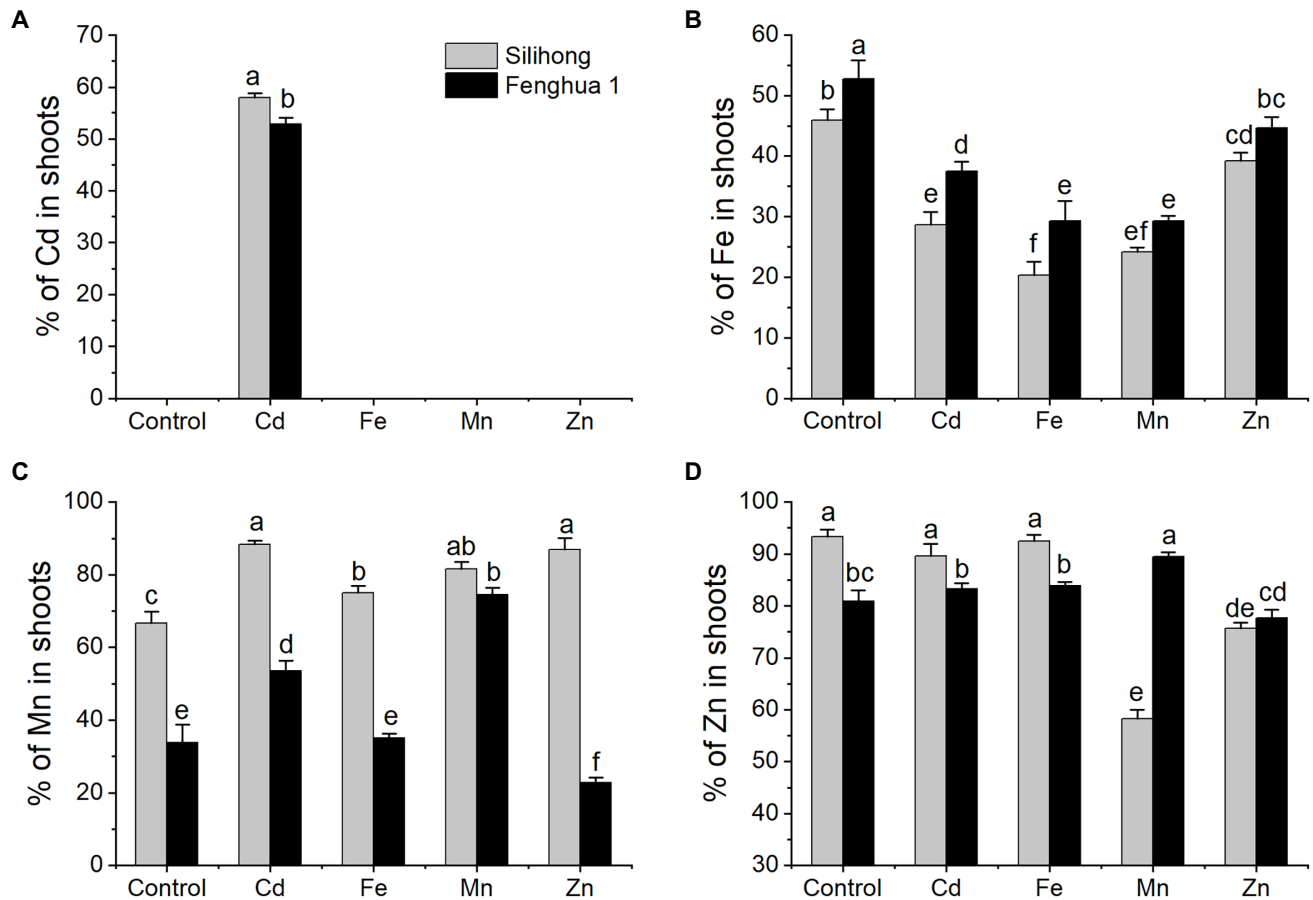
Percentages of Cd **(A)**, Fe **(B)**, Mn **(C)**, and Zn **(D)** in the shoot of two peanut cultivars exposed to excess metals. Data (means ± SE, *n* = 3) shared the same letter(s) above the error bars are not significantly different at the 0.05 level by Duncan multiple range test.

To investigate differences of metal translocation between the two peanuts, the percentage of metal in shoots was considered as an indicator for the translocation capability of metals from roots to shoots. Generally, Silihong exhibited higher capacity for the translocation of Cd and Mn from roots to shoots, while Fenghua 1 showed a higher capability of Fe translocation (Figures 6A–C). There are interactive effects between cultivar and metal treatment on the percentage of Mn (*F* = 30.34, *p* = 0.000) and Zn (*F* = 65.97, *p* = 0.000) in shoots, indicating that cultivar differences in the translocation of Mn and Zn from roots to shoots were dependent on metal exposure (Figures 6C,D). Exposure of excess Cd, Fe, Zn, and Mn significantly reduced Fe translocation from roots to shoots for both cultivars, which was more pronounced in Fe and Mn treatments. With regard to Mn, its translocation in Silihong was increased by all metal treatments, whereas in Fenghua 1, it was increased by Cd and Mn treatments but decreased by the Zn treatment. Exposure of excess Cd and Fe did not affect the translocation of Zn for both cultivars; however, it was decreased by the Zn treatment. Mn exposure dramatically reduced Zn translocation in Silihong but unaffected that in Fenghua 1. A significant and negative correlation was observed between the percentage of Fe and Mn in shoots (*r* = −0.498, *n* = 30, *p* = 0.005).

To determine whether *AhMTP* genes were involved in metal translocation in peanut plants, a stepwise linear regression analysis was performed on the percentage of metal in shoots and expression of *AhMTP* genes (−ΔΔCT). As showed in Table 6, the percentage of Fe in shoots significantly and positively correlated with the expression of *AhMTP4.1*, *AhMTP9.1*, and *AhMTPC4.1* but negatively correlated with that of *AhMTPC2.1* and *AhMTP12*. The expression of *AhMTP1.1* showed a significant and negative correlation with the percentage of Mn in shoots. The percentage of Zn in shoots was significantly and positively correlated with the expression of *AhMTP2.1* but was negatively correlated with that of *AhMTPC2.1*.

**Table 6:** Stepwise linear regression analysis of the percentage of metal in shoots and expression of *AhMTP* genes (*n* = 30).

Gene expression (−ΔΔCT)	% of Fe in shoots			% of Mn in shoots			% of Zn in shoots		
	*β*	*T*	*R*	*β*	*T*	*R*	*β*	*T*	*R*
*AhMTP1.1*	0.086	0.380	0.079	−0.445^*^	−2.627	−0.445	0.261	1.240	0.236
*AhMTP2.1*	−0.110	−0.678	−0.140	0.208	0.968	0.183	0.509^**^	2.978	0.497
*AhMTP4.1*	0.657^***^	4.168	0.648	0.098	0.487	0.093	−0.156	−0.755	−0.147
*AhMTP9.1*	0.810^***^	4.613	0.686	−0.145	−0.846	−0.161	−0.185	−1.049	−0.202
*AhMTP10.1*	−0.242	−1.515	−0.301	0.366	1.882	0.341	0.086	0.371	0.072
*AhMTP11.1*	−0.111	−0.632	−0.131	0.434	1.897	0.343	−0.096	−0.462	−0.090
*AhMTP12*	−0.976^**^	−3.196	−0.546	0.261	1.010	0.191	0.125	0.583	0.114
*AhMTPB1*	0.004	0.032	0.007	−0.118	−0.691	−0.132	−0.212	−1.366	−0.259
*AhMTPC2.1*	−1.751^***^	−7.252	−0.829	0.071	0.350	0.067	−0.579^**^	−3.385	−0.546
*AhMTPC4.1*	1.905^***^	5.590	0.752	−0.081	−0.398	−0.076	0.191	0.843	0.163

* *p* *< 0.05;*

** *p* *< 0.01;*

*** *p < 0.001*.

## Discussion

Genome-wide identification of MTP proteins has been extensively performed in diverse plant species, including *Arabidopsis* (Montanini et al., 2007), sweet orange (*Citrus sinensis*) (Fu et al., 2017), wheat (Vatansever et al., 2017), turnip (*Brassica rapa* var. *rapa*) (Li et al., 2018), tobacco (*Nicotiana tabacum*) (Liu et al., 2019), grape (*Vitis vinifera*) (Shirazi et al., 2019), and *Populus trichocarpa* (Gao et al., 2020). However, there is little information about MTP family in peanut that limits understanding the molecular mechanisms underlying the regulation of metal homeostasis. Herein, 24 putative *AhMTP* genes were identified in cultivated peanut, which were divided into seven groups (1, 5, 6, 7, 8, 9, and 12), belonging to three major substrate-specific groups (Zn-CDFs, Zn/Fe-CDFs, and Mn-CDFs) (Figure 1). Our findings concurred with the results obtained from *Arabidopsis* (Montanini et al., 2007), turnip (Li et al., 2018), tobacco (Liu et al., 2019), and *P. trichocarpa* (Gao et al., 2020), suggesting that AhMTPs may have similar functions to their homologs in these plant species.

In agreement with previous studies (Vatansever et al., 2017; Liu et al., 2019; Shirazi et al., 2019; Gao et al., 2020), most of AhMTP proteins were predicted to localize to vacuole membranes. All AhMTP proteins except AhMTP2.4 contained the typical domain of MTP, cation efflux domain. These results indicated that AhMTPs might be metal transporters playing key roles in the homeostasis or detoxification of divalent metals. Although AhMTP2.4 does not contain cation efflux domain, collinearity analysis revealed that it is a whole-genome duplicated gene of *AhMTP2.3* (Figure 2B). Moreover, phylogenetic analysis indicated that AhMTP2.4 shows a close relationship with other members of group 6 (Figure 1). Therefore, it could be regarded as an AhMTP member.

A total of 10 AhMTPs were identified to be Mn-CDF members, which were further divided into two groups: group 8 and 9. These proteins share the following characteristics: (1) containing both the cation efflux domain and ZT-dimer; (2) shared a specific motif complex composed of motifs 1, 2, 4, 5, and 9, among them motifs 4 and 5 were annotated to be the TMD and CTD of cation efflux, respectively; (3) containing four to six TMDs; (4) best modeled with 3h90.1 (Structural basis for the autoregulation of the zinc transporter YiiP); and (5) localizing to vacuolar or plasma membranes. YiiP is a dimeric Zn^2+^/H^+^ antiporter from *Escherichia coli* that selectively binds Zn^2+^ or Cd^2+^ to the active site and transports the bound ions across the bacterial membrane using the proton motive force (Lu et al., 2009; Lopez-Redondo et al., 2018). The presence of ZT-dimer and specific motif complex in Mn-CDFs suggested that these proteins might serve as metal transporters by forming homodimers or heterodimers functional complexes (Liu et al., 2019). These structural features indicate that Mn-CDFs might be multiple metal transporter in peanut plants.

Some proteins of group 8 have been proven to be vacuolar-or Golgi-localized Mn transporters (Delhaize et al., 2003; Chen et al., 2013; Pedas et al., 2014). Meanwhile, several members of group 9, including AtMTP11 from *Arabidopsis* (Delhaize et al., 2007; Peiter et al., 2007), OsMTP11 from rice (Tsunemitsu et al., 2018), and BmMTP10 and BmMTP11 from *Beta vulgaris* spp. *maritima* (Erbasol et al., 2013), were also characterized as the Golgi- and/or pre-vacuolar-localized transporters that confer Mn tolerance *via* intra-cellular Mn compartmentalization.

A total of eight Zn-CDF members were identified in peanut, which were further divided into three groups: group 1 (AhMTP1.1/1.2/1.3 and AhMTPB1/B2), 5 (AhMTPC2.1/C2.2), and 12 (AhMTP12). Unlike the Mn-CDF subfamily, Zn-CDF members share characteristics as follows: (1) containing cation efflux domain but not ZT-dimer; (2) containing motifs 6 and 11, which were annotated as cation efflux TMD; (3) containing five to six TMDs except AhMTP1.3 and AhMTP12, which contained 2 and 16 TMDs respectively; (4) best modeled with 6xpd.1 (Cryo-EM structure of human ZnT8 double mutant—D110N and D224N, determined in outward-facing conformation); and (5) localizing to vacuolar membranes. ZnT8 is a Zn^2+^/H^+^ antiporter that plays essential roles in regulating Zn^2+^ accumulation in the insulin secretory granules of pancreatic β cells (Xue et al., 2020). The structural features suggest that Zn-CDF members might serve as Zn transporters in peanut plants. Two Zn-CDF members from *Arabidopsis*, AtMTP1 and AtMTP3, belonging to group 1, have been showed to be Zn transporters that are localized to vacuolar membrane and mediate the vacuole sequestration of Zn (Kobae et al., 2004; Arrivault et al., 2006).

Interestingly, AhMTP12 contained 16 TMDs, which is higher than the homologous gene from other plant species, including BrrMTP12 from turnip (Li et al., 2018), CitMTP12 (12 TMDs) from sweet orange (Fu et al., 2017), PtrMTP12 (12 TMDs) from *P. trichocarpa* (Gao et al., 2020), VvMT12 (12 TMDs) from grape (Shirazi et al., 2019), and NtMTP12.1 (10 TMDs) and NtMTP12.2 (eight TMDs) from tobacco (Liu et al., 2019). It was also observed that AhMTP12 had the largest protein size (867 amino acids) and MW (97.04 kDa). The results were in accordance with the characteristics of MTP12 in other plants (Li et al., 2018; Liu et al., 2019; Gao et al., 2020). Moreover, collinearity analysis showed that *AhMTP12* is the only gene in the *AhMTP* family that has not experienced gene duplication events. The current results indicate that *AhMTP12* might be distinctive both in the evolutionary process and physiological functions in peanut plants. In *Arabidopsis*, AtMTP12 has been shown to forms a functional complex with AtMTP5t1, one of the splicing variants of AtMTP5, transporting Zn into the Golgi (Fujiwara et al., 2015).

Another distinctive gene of Zn-CDFs is *MTP1.3*, which has the shortest sequence of the gene and protein. Only two TMDs and two motifs (6 and 11) were harbored in the MTP1.3 protein. Collinearity analysis revealed that *AhMTP1.3* and *AhMTP1.2* might be resulted from segmental duplication within the same subgenome.

The six Zn/Fe-CDF members were divided into two significantly different groups: Group 6 (AhMTP2.1/2.2/2.3/2.4) and 7 (AhMTPC4.1/C4.2). The two members of group 7 are WGD genes, which have the following common characteristics: (1) containing cation efflux domain but not ZT-dimer; (2) only containing two motifs (11 and 15), of which motif 11 were annotated as cation efflux TMD; and (3) localizing to vacuolar membranes. In contrast, the group 6 is composed of two pairs of WGD genes (*AhMTP2.1/2.2* and *AhMTP2.3/2.4*). The pair of *AhMTP2.1/2.2* is similar in all structural characteristics both at protein and gene levels. However, there are significant differences in the structure of the gene and protein between *AhMTP2.3* and *AhMTP2.4*. For instances, while AhMTP2.3 contained six TMDs, AhMTP2.4 only contained three TMDs. Unlike AhMTP2.1/2.2 that contained both cation efflux domain and ZT-dimer, AhMTP2.3 contained cation efflux domain but not ZT-dimer, whereas AhMTP2.4 contained ZT-dimer but not cation efflux domain. A 3D-model prediction revealed that all Zn/Fe-CDFs were commendably modeled to 5vrf.1 except AhMTP2.4, which preferentially modeled with 3h90.1. The large divergence in the protein structure suggested that AhMTP2.3 and AhMTP2.4 might have distinctive physiological functions in peanut plants.

Gene duplication as a major source of novel genes contributes to the acquirement of novel functions (Panchy et al., 2016). Peanut is an allotetraploid species whose genome contains essentially complete sets of chromosomes (AABB) from two diploid ancestral species, *A. duranensis* (AA) and *A. ipaensis* (BB) (Bertioli et al., 2019). Expectedly, our results indicate that almost all *AhMTP* genes experienced gene duplication events except *AhMTP12*, resulting in 13 gene pairs (Figure 2B). Among them, 11 gene pairs were evolved from WGDs, and *AhMTP1.3* and *AhMTP1.2* might be segmentally duplicated genes. Gene pairs of duplication were consistent with the results obtained from phylogenetic analysis.

Duplicated genes, if they survive, would diverge in both the regulatory and coding regions during evolution, resulting in distinct functions (Xu et al., 2012). Thus, exon-intron structure can provide additional evidence to support phylogenetic analysis of gene families (Zhang et al., 2012). In the present study, we found that *AhMTP* genes within the same phylogenetic group show highly similar exon-intron structures, despite there are large variations among groups even in the same subfamily (Figure 2A). While most of the WGD gene pairs shared a similar exon-intron structure, divergences were also found within three gene pairs (*AhMTPC4.1/C4.2*, *AhMTP2.3/2.4*, and *AhMTP9.1/9.2*). Divergences of duplicate genes in exon-intron structure mainly resulted from three mechanisms: exon-intron gain/loss, exonization/pseudoexonization, and insertion/deletion (Xu et al., 2012). Although all the gene pairs experienced exon-intron gain/loss events, exonization/pseudoexonization also occurred in the evolutionary process of *AhMTP2.3/2.4* and *AhMTP9.1/9.2*, which might contribute to functionally distinct paralogues.

It is noteworthy that six Zn-CDF genes belonging to group 1 (*AhMTP1.1/1.2/1.3* and *AhMTPB1/B2*) and 12 (*AhMTP12*) have one exon without intron in CDS, which is called single exon genes (SEG) (Sakharkar et al., 2004). The presence of SEG in multi-cellular eukaryotic genomes intriguing since such kind of genes are archetypal of prokaryotes (Sakharkar et al., 2004). SEGs can be divided into two groups: (i) UTR intron-containing SEGs (uiSEGs) that have introns in their untranslated region (UTR) and (ii) intronless genes (IGs) lacking introns in the entire gene (Jorquera et al., 2018). Herein, *AhMTP1.3* having an intron in the UTR belong to uiSEGs while *AhMTP1.1/1.2*, *AhMTPB1/B2*, and *AhMTP12* are IGs. SEGs have been reported in MTP family from tobacco (Liu et al., 2019) and tomato (*Solanum lycopersicum*) (El-Sappah et al., 2021).

Gene expression was generally regulated at two different levels: transcriptional and post-transcriptional. CRE play essential roles in the transcriptional regulation of gene expression by interacting with specific transcription factors and RNA polymerase. In the current study, a large number of CAAT-box and TATA-box were detected in the promoters of *AhMTP* genes; this is expectedly because the two elements are involved in the regulation of expression frequency and initiation of transcription (Laloum et al., 2013). Besides, the wide distribution of light-responsive elements, phytohormonal responsive elements, and abiotic stress in the promoters indicate that expression of *AhMTP* genes could be regulated by many factors in multiple pathways. MYB-binding sites (MBS) were proposed to be involved in metal tolerance by driving *MTP1* expression in *Arabidopsis helleri* (Fasani et al., 2017). The location of MBS in the promoters of *AhMTP1.1/1.2*, *AhMTPC2.1*, *AhMTP12*, and *AhMTP4.1* suggests that these genes might be regulated at the MYB-binding sites.

MicroRNAs are generally believed to downregulate the expression of target genes by cleaving mRNA or inhibiting the translation of target genes (Bartel, 2009). Four pairs of *AhMTP* genes (*AhMTP9.1/9.2*, *AhMTPC2.1*/*C2.2*, *AhMTP4.1*/*4.2*, and *AhMTP11.2/11.3*) were predicted as targets of five miRNAs including *ahy-miR167-5p*, *ahy-miR156a*, *ahy-miR156c*, *ahy-miR3511-3p*, and *ahy-miR3519*. Although the function of *miR3511-3p* and *miR3519* is unknown, the regulation of *miR167* and *miR156* on their targets has been extensively studied. The *miR167* has been reported to target the mRNAs encoding the *ARF6*, *ARF8*, and *IAR3*, regulating auxin signaling and homeostasis in *Arabidopsis* (Wu et al., 2006; Kinoshita et al., 2012; Yao et al., 2019). Another study demonstrated that *BnNRAMP1b* is a target of *miR167* in *Brassica napus* (Meng et al., 2017). *MiR156a* and *miR156c* play dominant roles in regulating abiotic stress resistance through a *miR156-SPL* regulatory pathway (Cui et al., 2014; Wang et al., 2021). The expression of *miR167* and *miR156* was downregulated by Ca deficiency in peanut embryos (Chen et al., 2019). Similar results were reported in the roots and shoots of high-Fe rice line under Fe deficiency (Agarwal et al., 2015). Together, it can be inferred that *AhMTP9.1/9.2* and *AhMTPC2.1*/*C2.2* might be post-transcriptionally repressed by *ahy-miR167-5p* and *ahy-miR156*(a/c), respectively, which deserves to be experimentally tested.

Tissue expression profiles indicate that half of *AhMTP* genes showed low expression in the 22 tissues, among them, six genes (*AhMTP2.3*/*2.4*, *AhMTPB1*/*B2*, *AhMTP11.4*, and *AhMTP12*) were not or rarely expressed in any tissues. The reduced gene expression of these genes might be beneficial for maintenance of their biological functions and avoiding gene loss during evolution processes (Qian et al., 2010; Liu et al., 2019). With respect for the remaining 18 *AhMTP* genes, most of them preferentially expressed in reproductive tissues such as stamens, peg tips, fruit, peg tip to fruit, pericarp, and seed. These genes might play roles in the development of pods or seeds by mediating the transport of divalent metals. High expression levels of *AhMTP10.1*/*10.2* in leaves, perianth, and pistil indicate they might be involved in metal homeostasis in leaves and flower.

Due to most of AhMTP proteins were predicted to localize to vacuole membranes, we speculated that changing expressions of *AhMTP* genes in roots might alter the root-to-shoot translocation of divalent metals. To test this hypothesis, the expression of *AhMTP* genes in roots of two peanut cultivars in response to metal stresses and its relationship to metal translocation was investigated. *AhMTP* genes showed wide differences in response to various metal stresses depending on cultivars and metal exposure. Generally, 10 *AhMTP* genes tested in the current study were sensitive to excess metal exposure and most of them showed higher expressions under metal stress (particularly in Fe, Cd, and Zn treatments). In agreement with previous studies (Liu et al., 2017; Yu et al., 2019), we found that Silihong showed higher capacity for the translocation of Cd from roots to shoots than Fenghua 1. Cd translocation was also reported to be decreased by increasing Cd concentration in rhizosphere (Yu et al., 2019). Most of *AhMTP* genes were induced by Cd in a cultivar-dependent manner, and the increase of gene expression was more pronounced in Silihong than in Fenghua 1. These results indicate that *AhMTP* genes, particularly *AhMTP1.1*, *AhMTPB1*, *AhMTPC2.1*, and *AhMTP10.1*, might be responsible for the cultivar difference of Cd translocation in peanuts.

Stepwise linear regression analysis revealed Fe translocation from roots to shoots might be associate to *AhMTP4.1*, *AhMTP9.1*, *AhMTPC4.1*, *AhMTPC2.1*, and *AhMTP12*. The upregulation of *AhMTPC2.1* and *AhMTP12* might contribute to the reduction of Fe translocation *via* sequestration of Fe in vacuoles or other vesicles under excess metal exposure. More sensitive response of *AhMTP4.1* and *AhMTPC4.1* to metal exposure in the roots might be responsible for the higher translocation of Fe in plants of Fenghua 1.

The significant and negative correlation of the expression of *AhMTP1.1* and the percentage of Mn in shoots indicates that *AhMTP1.1* might be involved in Mn translocation. The downregulation of *AhMTP1.1* in the root under Fe, Mn, and Zn stresses might contribute to increased Mn translocation in Silihong. Higher Mn translocation in Fenghua 1 under metal exposure might result from higher expression of *AhMTP1.1* in the root. The translocation of Zn was not affected by Cd and Fe exposure, while excess Zn reduced its translocation in Silihong, and excess Mn largely decreased Zn translocation in Silihong but increased that in Fenghua 1. Stepwise linear regression analysis revealed *AhMTP2.1* and *AhMTPC2.1* might be associate with Zn translocation in peanut plants. Thus, the reverse change of percentages of Zn in shoots between Silihong and Fenghua 1 under Mn exposure might also be in part a consequence of the reverse response of *AhMTP2.1* and *AhMTPC2.1* to Mn exposure in the root of the two cultivars.

## Conclusion

A total of 24 *AhMTP* genes were identified in peanut, which were divided into seven groups belonging to three substrate-specific clusters (Zn-CDFs, Zn/Fe-CDFs, and Mn-CDFs). All *AhMTP* genes underwent whole genome or segmental gene duplication events except *AhMTP12*. Most *AhMTP* members within the same subfamily or group generally have similar gene and protein structural characteristics. However, some genes, such as *AhMTP1.3*, *AhMTP2.4*, and *AhMTP12*, showed wide divergences. Most of *AhMTP* genes preferentially expressed in reproductive tissues, suggesting that these genes might play roles in metal transport during the pod and seed development stages. Excess metal exposure induced expressions for most of *AhMTP* genes in peanut roots depending on cultivars. By contrast, *AhMTP* genes in the root of Fenghua 1 were more sensitive to excess Fe, Cd, and Zn exposure than that of Silihong. The differential responses of *AhMTP* genes to metal exposure might be, at least partially, responsible for the different metal translocation from roots to shoots between Fenghua 1 and Silihong. The findings provide clues to further characterize the functions of AhMTP proteins in the uptake and translocation of metal ions in peanut plants, which is great of importance for screening or breeding cultivars for peanut safe production in heavy metal contaminated soil.

## Data Availability Statement

The datasets presented in this study can be found in online repositories. The names of the repository/repositories and accession number(s) can be found at: NCBI’s GEO database (https://www.ncbi.nlm.nih.gov/geo/query/acc.cgi?acc=GSE71357), accession number: GSE71357.

## Author Contributions

XW and CW carried out most of the experimental work with assistance from GS and ZZ. GS and ZZ were responsible for experimental design. GS and XW carried out data analyses and wrote and revised the manuscript. All authors contributed to the article and approved the submitted version.

## Funding

This work was supported by grants from the Natural Science Foundation of Anhui Province (grant number 2108085MC83), the Natural Science Foundation for Colleges and Universities of Anhui Province (grant numbers KJ2020ZD83 and KJ2019A0587), and the Innovation Team of Scientific Research Platform of Anhui Province (grant number KJ2015TD001).

## Conflict of Interest

The authors declare that the research was conducted in the absence of any commercial or financial relationships that could be construed as a potential conflict of interest.

## Publisher’s Note

All claims expressed in this article are solely those of the authors and do not necessarily represent those of their affiliated organizations, or those of the publisher, the editors and the reviewers. Any product that may be evaluated in this article, or claim that may be made by its manufacturer, is not guaranteed or endorsed by the publisher.

## Supplementary Material

The Supplementary Material for this article can be found online at: https://www.frontiersin.org/articles/10.3389/fpls.2022.791200/full#supplementary-material

## Footnotes

^1^ https://phytozome-next.jgi.doe.gov

^2^ https://peanutbase.org

^3^ https://www.ebi.ac.uk/Tools/hmmer/search/hmmscan

^4^ https://itol.embl.de/itol.cgi

^5^ https://web.expasy.org/protparam/

^6^ http://topcons.net/

^7^ http://www.csbio.sjtu.edu.cn/bioinf/plant-multi/

^8^ https://meme-suite.org/meme/tools/meme

^9^ http://pfam.xfam.org/search#tabview=tab1

^10^ http://www.swissmodel.expasy.org

^11^ http://gsds.gao-lab.org/

^12^ https://www.peanutbase.org/gene_expression/atlas

## Abbreviations

CDF: Cation diffusion facilitator
CDS: Coding sequence
Chr: Chromosomes
GRAVY: Grand average of hydropathicity
FPKM: Fragments per kilobase of exon model per million mapped reads
Ka: The number of nonsynonymous substitutions per nonsynonymous site
Ks: The number of synonymous substitutions per synonymous site
MTP: Metal tolerance protein
MW: Molecular weight
pI: Isoelectric point
qRT-PCR: Quantitative real time PCR
TMDs: Transmembrane domains
