# Supplementary material for: Genome-wide Identification of Metal Tolerance Protein Genes in Peanut: Differential Expression in the Root of Two Contrasting Cultivars Under Metal Stresses
Source: Front Plant Sci. 2022 Apr 1;13:791200. doi: 10.3389/fpls.2022.791200 (PMC9011049; doi:10.3389/fpls.2022.791200)
Supplement: Supplementary file 2 [file Table_1.DOCX]

**Supplementary Figure 1**. Predicted 3D structure of peanut AhMTP proteins by SwissModel. Models were visualized by rainbow color from N to C terminus.
